# Exploring the Origin of Cells in the Progression of Primary Myelofibrosis to Acute Myeloid Leukemia Case Report

**DOI:** 10.1155/crh/5472609

**Published:** 2026-06-09

**Authors:** Philip Papayanis, Kara Conroy, Melissa Larson, Sunita Nathan, Celalettin Ustun

**Affiliations:** ^1^ Department of Medicine, Division of Internal Medicine, Rush University Medical Center, Chicago, Illinois, USA, rush.edu; ^2^ Department of Medicine, Division of Hematology, Oncology and Cellular Therapy, Rush University Medical Center, Chicago, Illinois, USA, rush.edu

**Keywords:** acute myelogenous leukemia, case report, cell lineage, primary myelofibrosis

## Abstract

Primary myelofibrosis (PMF) is the clonal proliferation of common myeloid progenitor (CMP) cells and can transform into acute myeloid leukemia (AML). The exact transformation process and cell lines involved are not well‐understood. A patient with PMF received an allogenic hematopoietic stem cell transplant (alloHCT) from his major ABO incompatible sibling and developed pure red cell aplasia that resolved when his blood type switched to that of his donor’s. He relapsed with AML 10 months after alloHCT; interestingly, his blood type remained his donor’s type. This supports that AML in this patient with PMF stemmed from more committed progenitors (myeloblasts) than CMP.

## 1. Introduction

Primary myelofibrosis (PMF), a myeloproliferative neoplasm (MPN), involves clonal proliferation of pluripotent myeloid progenitor stem cells. Patients with PMF typically have abnormalities in platelet, white blood cell (WBC), and red blood cell (RBC) counts or function [1]. PMF can evolve into acute myeloid leukemia (AML), a clonal hematopoietic malignancy marked by the accumulation of immature myeloid blasts [2, 3]. This evolution occurs through clonal changes, with a 10‐year risk of 5.8%–20.6% [1]. While evolution is somewhat common, the specific progenitor cells involved remain unclear. We present a patient with PMF who progressed to AML after allogeneic hematopoietic stem cell transplantation (alloHCT) with some interesting post‐transplant findings that provide insight into cell lineage.

## 2. Case Presentation

A 54‐year‐old male presented to the hematology clinic for anemia late in 2023. His complete blood count (CBC) was notable for hemoglobin (Hb) 7.3 g/dL, reticulocytes 1.3%, platelets 451 k/uL, and WBC 5.24 k/uL, with a differential of 55% neutrophils, 3% metamyelocytes, 1% myelocytes, 27% lymphocytes, 5% monocytes, 4% basophils, and 2% other cells. His bone marrow biopsy showed a hypercellular marrow (100%) with 3% blasts, increased atypical megakaryocytes, granulocyte hyperplasia, and Grade 2‐3 fibrosis, consistent with an early fibrotic phase PMF. Genetic testing did not show any mutations in *BCR/ABL*, *JAK2*, *MPL*, or *CALR*; however, *ASXL1* and *SF3B1* were mutated. His karyotype was normal. He was diagnosed with MPN, most likely PMF, and had a clinical course of severe, transfusion‐dependent, luspatercept‐refractory anemia. Despite having intermediate‐risk DIPSS [3] and MIPSS70 [4] scores, he received peripheral blood stem cells from his HLA‐fully matched 59‐year‐old brother following reduced‐intensity conditioning (fludarabine and melphalan) in May 2024 because of severe anemia. His graft versus host disease (GvHD) prophylaxis was tacrolimus and mini‐methotrexate.

His transplantation course was remarkable for pure red cell aplasia (PRCA) due to major ABO incompatibility (donor’s blood type A Rh+, recipient’s O Rh+). Pretransplant isoagglutinin A IgG and IgM titers were elevated to 1:64 and 1:32, respectively. Due to the refractory nature of his disease and significant symptomatic anemia (Hb 4‐5 with reticulocyte 0%–0.1%), multiple therapies, including plasmapheresis, rituximab, eltrombopag, and steroids, were used, as well as a donor lymphocyte infusion (DLI), luspatercept, and bortezomib. Gradual Hb and reticulocyte recovery began about 8 months post‐transplant, coinciding with conversion to donor blood type (A Rh+) and decreased isoagglutinin A IgG and IgM titters (1:4 and 1:8, respectively). By 2 months post‐transplant, he had 100% donor chimerism in the myeloid and T cell lineages in peripheral blood. While his immunosuppression was tapered, he developed thrombocytopenia, and donor chimerism decreased (whole blood 96%, T cell 98%, and myeloid 98%). A repeat bone marrow biopsy 10 months post‐transplant showed 50% cellularity with 40% myeloid blasts, Grade 2 fibrosis, and 68% donor chimerism. Overall, his pathology findings revealed an unexpected progression to AML. Genetic testing again showed *ASXL1* and *SF3B1* mutations, but not any new genetic abnormalities. Ultrasound did not reveal splenomegaly. His blood type remained A Rh + instead of reverting to O+, confirmed repeatedly by the blood bank. He received an AML‐type induction chemotherapy and achieved his second complete response (CR2) with 100% donor chimerism shortly afterwards. He received A Rh + RBC units without any reactions, confirming his donor blood type persisted. His bone marrow biopsy in April 2025 confirmed this with only 5% cellularity but no residual blasts Grade 2 fibrosis. His Hb recovered quickly with no evidence of hemolysis or PRCA (again confirming his blood type remained A Rh+). A second DLI was given to prevent further relapses. He remained in remission on maintenance azacitidine with a fully recovered CBC until he unfortunately developed severe liver GVHD and passed away 4 months after his second DLI.

## 3. Discussion

**FIGURE 1:**
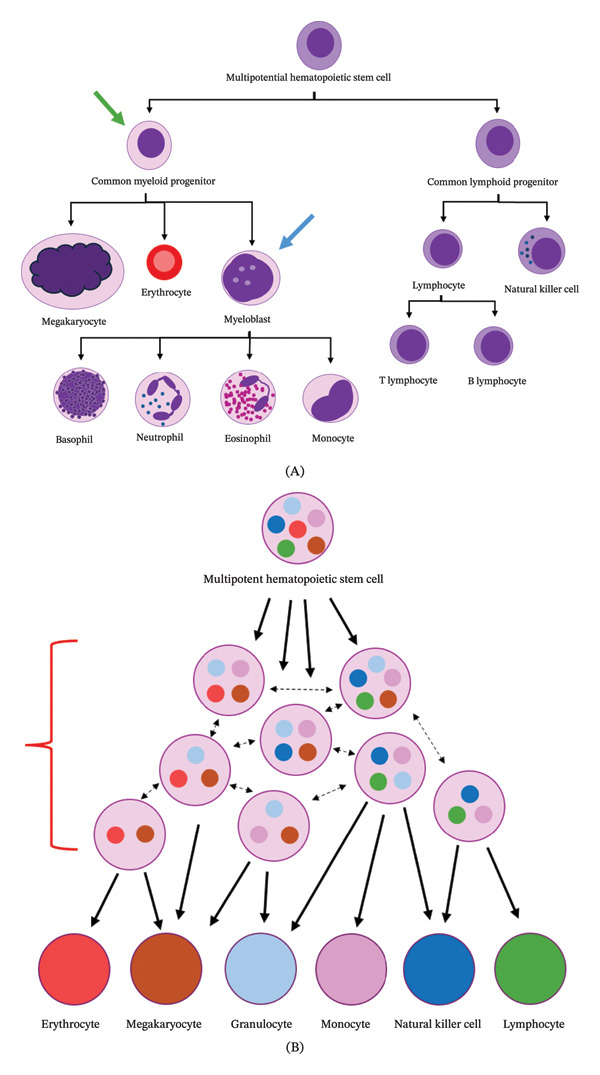
Hypothetical cell lineage for our patient. (A) Proposed lineage under the classical model. The green arrow denotes the monoclonal cell in MPNs (such as our patient’s PMF). The blue arrow denotes the suspected monoclonal cell in our patient’s AML. This illustrates how we suspect our patient’s PMF transformed into AML but became a more mature cell line which is unexpected. (B) Representation of the continuum model of immune cell differentiation where cells acquire lineage bias simultaneously before fully differentiating. The red bracket denotes cells which have some lineage differentiation but still have multiple potential effector cells they could develop into and represent what we suspect our patient’s malignancy developed from (both figures are simplified for illustrative purposes and are not meant to capture all the complexities of hematopoietic cell lineages).

Relapses of PMF after alloHCT are a known complication, with several studies demonstrating a rate around 20% [4, 5]. Relapse typically presents with gradually declining counts, slight increases in myeloblasts, and splenomegaly. Direct progression to AML after alloHCT, as observed in our patient, is much rarer, with no clear rate of transformation. One study reported that < 3% of patients who received alloHCT for PMF developed leukemia [6]. Interestingly, our patient’s RBC lineage remained his donor’s type despite his myeloid leukemia originating from recipient cells. This supports the theory that the progression did not stem from the patient’s original multilineage hematopoietic progenitor stem cell (i.e., common myeloid progenitors) but from more mature, committed uni‐lineage progenitor cells (i.e., myeloblasts) per the classical lineage tree model (Figure 1A). This was very interesting and unexpected, but notably this theory has a limitation since lineage‐specific data such as clonal evolution or chimerism analysis were not obtained for our patient which limits the ability to truly determine the origin of his relapsed cells. There are newer models of clonal evolution (i.e., lineage continuum model) suggesting that rather than having clear progression with discrete hierarchical stages, hematopoietic stem cells exist on a continuum and gradually acquire different lineage biases simultaneously before differentiating into an effector cell [7, 8] (Figure 1B). Per this model, it is possible that both his PMF and AML in our case originated from a similar linage in terms of differentiation and that that lineage in relapse simply did not overlap with RBCs on the continuum. The differentiation bias could be different at diagnosis and at relapse due to some external factors affecting the same/similar oligopotent cells, such as growth factors. The patient only received granulocyte‐colony stimulating factor (G‐CSF) before engraftment (about 8 months before transformation); therefore, it was unlikely to be the cause of relapse as AML. The patient received erythropoietin (EPO) for 1 month and eltrombopag for 4 months; however, their role in disease evolution remains unclear. The risk of using eltrombopag in MDS is not clear, as evidenced by a Phase III trial showing an increased risk of progression from MDS to AML in patients with intermediate and high‐risk MDS who received azacitidine [9], but another Phase II trial in high‐risk MDS and a Phase II trial in low‐risk MDS showed no increased risk of progression [10, 11]. Likewise, EPO has not been found to increase the risk of progression in MDS [12]. In our patient, both EPO and eltrombopag were stopped about 3 months before his relapse. PRCA treatments are not generally associated with progression to AML (e.g., luspartecept) [13], plasmapheresis, bortezomib, steroids, and rituximab). In addition to no clear iatrogenic cause for the transformation, there were no new genetic aberrations. Moreover, the transformation occurred while he was on much less immunosuppression compared to the initial months after alloHCT.

In conclusion, we believe this case sheds some light on the cell origin of AML in the progression of MPNs, suggesting that his AML developed from more committed progenitor cells rather than his original progenitor cells of MPN, though exact cell origin cannot be confirmed; clonal origin testing was not performed. Our patient’s progression also happened with no clear predisposing factor.

## Author Contributions

Philip Papayanis and Celalettin Ustun: conceived the concept, literature search, collecting data, and writing and editing the manuscript.

Kara Conroy, Melissa Larson, and Sunita Nathan: literature search, collecting data, and writing and editing the manuscript.

## Funding

Any publication fees will be funded by Rush University Department of Hematology, Oncology, and Cellularly Therapy.

## Ethics Statement

In order to minimize harm to the patient this article had all identifying information removed.

## Consent

The patient has provided written consent to the writing and publication of this case report.

## Conflicts of Interest

The authors declare no conflicts of interest.

## Data Availability

Nonidentifiable information about the case can be provided if requested.
